# Gene expression of fatty acid binding protein genes and its relationship with fat deposition of Thai native crossbreed chickens

**DOI:** 10.5713/ajas.20.0020

**Published:** 2020-04-13

**Authors:** Supanon Tunim, Yupin Phasuk, Samuel E. Aggrey, Monchai Duangjinda

**Affiliations:** 1 Department of Animal Science, Faculty of Agriculture, Khon Kaen University, Khon Kaen, 40002, Thailand; 2 Research and Development Network Center for Animal Breeding (NCAB), Faculty of Agriculture, Khon Kaen University, Khon Kaen, 40002, Thailand; 3 NutriGenomics Laboratory, Department of Poultry Science, University of Georgia, Athens, GA 30602, USA

**Keywords:** Gene Expression, Fatty Acid Binding Proteins (FABPs), Fat Deposition, Correlation, Crossbreed Chicken

## Abstract

**Objective:**

The objectives of this study were to investigate the relationship between the mRNA expression of adipocyte type fatty acid binding protein (A-FABP) and heart type FABP (H-FABP) in Thai native chicken crossbreeds and evaluate the level of exotic inclusion in native chicken that will improve growth while maintaining its relatively low carcass fat.

**Methods:**

The fat deposition traits and mRNA expression of A-FABP and H-FABP were evaluated at 6, 8, 10, and 12 weeks of age in 4 chicken breeds (n = 8/breed/wk) (100% Chee breed [CH] [100% Thai native chicken background], CH male and broiler female [Kaimook e-san1; KM1] [50% CH background], broiler male and KM1 female [Kaimook e-san2; KM2] [25% CH background], and broiler [BR]) using abdominal fat (ABF) and muscular tissues.

**Results:**

The BR breed was only evaluated at 6 weeks of age. At week 6, the CH breed had a significantly lower A-FABP expression in ABF and intramuscular fat (IF) compared with the other breeds. At 8 to 12 weeks, the KM2 groups showed significant upregulation (p<0.05) of A-FABP in both ABF and IF compared to the CH and KM1 groups. The expression of H-FABP did not follow any consistent pattern in both ABF and IF across the different ages.

**Conclusion:**

Some level of crossbreeding CH chickens can be done to improve growth rate while maintaining their low ABF and IF. The expression level of A-FABP correlate with most fat traits. There was no consistency of H-FABP expression across breed. A-FABPs is involved in fat deposition, genetic markers in these genes could be used in marker assisted studies to select against excessive fat accumulation.

## INTRODUCTION

Health problems are a global issue. A significant leading cause of mortality in the population is non-communicable disease (NCD) partially caused by overweight and obesity [1]. The change in daily diet with greater consumption of dense energy food containing high portion of dietary fat has been well known to enhance adverse effects on health and subsequently lead to NCDs [2]. Animals contain a greater proportion of fat when compared to other dietary sources [3]; therefore, the consumer often avoids excessive fat and considers it as an unwanted part of the diet. From an animal breeding viewpoint, excessive fat deposition should be selected against to respond to the desires of healthy consumers and improve feed efficiency. Compared to exotic breeds, Thai native chickens have been found to have low fat and excellent meat quality [4].

Unfortunately, production of Thai native chicken on a commercial scale is limited by their slow growth rate. The production of Thai native chicken on a commercial scale is usually undertaken through crossbreeding of the Thai native chickens with exotic breeds to increase growth efficiency. However, this approach also results in increased fatness of the crossbreeds. Therefore, genetic improvement requires breeding strategies to increase growth rate, while maintaining the advantages of low fat and meat quality. Zerehdaran et al [5] reported that selection programs to improve growth performance increases carcass fat deposition because of the positive genetic correlation between growth and fat deposition. Chickens used in this study were the Chee breed (CH) developed by the Research and Development Network Center for Animal Breeding, Khon Kaen University, Thailand. CH is a purebred lean native chicken with a white feather color. In addition, it was created by crossbreeding with a different breed fraction including 50% of Thai native Kaimook e-san1 (KM1) and 25% as Kaimook e-san2 (KM2).

Molecular genetics is an effective approach to increase progress in breeding by taking advantage of key genes shown to control fat deposition. Fatty acid binding proteins (FABPs) are proteins related to both extracellular and intracellular metabolism of lipids [6,7]. FABPs are functional genes relating to energy expenditure by using fat as source such as heart type fatty acid binding proteins (H-FABP or FABP3) [8,9]. Also part of the family is adipocyte type fatty acid binding protein (A-FABP or FABP4) related to energy storage [6,10].

The FABPs play a role in the transport of lipids to specific compartments in the cell: to lipid droplets for storage; to the endoplasmic reticulum for signaling, trafficking and membrane synthesis; to the mitochondria or peroxisome for oxidation; to the nucleus for the control of lipid-mediated transcriptional programs via nuclear hormone receptors [11]. Shi et al [12] reported that the A-FABP gene may affect lipid metabolism through peroxisome proliferator-activated receptor gamma. The mRNA expression of H-FABP has been shown to be negatively correlated with intramuscular fat in chicken breast and leg [13]. However, the gene expression information of Thai native chicken along with its crossbreeds, may different with other breeds, is limited. Teltathum and Mekchay [14] have also shown a negative relationship between both mRNA and protein expressions of FABP3 in the *Pectoralis* muscle of the Thai Praduhangdum chicken as it ages. From this viewpoint, we assumed that FABPs possibly affect the development of other tissues including adipose tissue. For these reasons, our study focuses on the most likely gene related to fat metabolism, so both A-FABP and H-FABP were investigated at the expression level in both muscular and adipose tissue.

The objective of this study was to investigate the relationship between the mRNA expressions of A-FABP and H-FABP in Thai native chicken crossbreeds and evaluate the level of exotic inclusion in native Thai chicken that will improve growth while retaining its relatively low carcass fat.

## MATERIALS AND METHODS

### Animals and management

#### Animals

All animals used in this study have been approved by Institute of Animal for Scientific Purpose Development (IAD, IACUC-KKU-34/62). Purebred and crossbreed Thai native chickens were received from Research and Development Network Center for Animal Breeding of Khon Kaen university, while Arbor Acres broilers was obtained from Charoen Pokphand Group Company Limited. The chicken genotypes used included CH (100% Thai native chicken background: 0% broiler background), CH ♂ and broiler ♀ (KM1) (50% Thai native chicken background: 50% broiler background), broiler ♂ and KM1 ♀ (KM2) (25% Thai native chicken background: 75% broiler background), and broiler (BR) (0% Thai native chicken background: 100% broiler background). A hundred birds of each breed were used with randomized sex and were individually tagged with wing bands.

#### Housing and management

All birds were reared under the same management and husbandry conditions and fed with commercial broiler diet throughout the experiment. The poultry house was an open-air system with stirring fan. The birds were fed *ad libitum* with feed including starting phase (21% crude protein (CP), 3% crude fiber (CF), and 3,100 kcal of ME/kg) for 1 to 3 weeks of age and growing phase (19% CP, 3% CF and 3,200 kcal of ME/kg) thereafter until slaughtering.

### Data and tissue collection

#### Fat deposition in carcass

Twenty birds (10 males and 10 females) of each breed were randomly selected and slaughtered at 6, 8, 10, and 12 weeks of age for CH, KM1, and KM2, while BR was slaughtered only at 6 weeks of age. The broilers were raised only till 6 weeks as they had become too large. All of sampling birds in each week were slaughtered; after that, the abdominal fat and skin (except wing tip skin) were separated from the chicken carcass and calculated as percentage of carcass weight.

#### Tissue collection

During the slaughtering process at 6, 8, 10, and 12, the major breast muscle (*Pectoralis* (*P.*) *major*) and abdominal fat of birds (4 males and 4 females) in each breed were immediately collected and placed into storage and snap frozen and stored at −20°C. Intramuscular fat was extracted from the *P. major*.

### Quantitative reverse transcription polymerase chain reaction

#### RNA extraction

Within 48 hours after slaughtering, total RNA was extracted from frozen abdominal fat tissue using GeneJET RNA Purification Kit (Thermo Scientific, Waltham, MA, USA) according to the manual and instruction protocol. The NanoDrop 2000/2000c Spectrophotometer (Thermo Scientific, USA) was used to determine the quality and quantity of total extracted RNA and the RNA was stored at −20°C until use as a template for transcriptional gene expression analysis.

#### qRT-PCR

Quantitative reverse transcription polymerase chain reaction (qRT-PCR) was performed using Bio-Rad CFX96 Touch Real-Time PCR Systems (Bio-Rad, Hercules, CA, USA), on optical grade plates using IQTM PCR plate (Bio-Rad, USA) to measure the transcriptional levels of *A-FABP* and *H-FABP* genes, and 18S ribosomal RNA (18S rRNA) was used as a reference gene. The SensiFAST SYBR No-ROX One-Step Kit (Bioline, Memphis, TN, USA) was used to investigate target RNA expression with one step RT-PCR. Primer sequences are reported in Table 1 and were purchased from 1st BASE Oligonucleotide Synthesis (1st Base, Singapore). Quantitative PCR was accomplished in duplicate for each sample.

#### Statistical analysis

The relative expression of *A-FABP* and *H-FABP* genes of each mRNA was normalized to the 18s rRNA gene using the 2^−ΔΔCt^ method [15]. The *FABP* genes transcription levels were reported as the fold change difference to the Thai native chickens (CH) which described as: ΔΔCt = (Ct_target_ – Ct_control_)_target breed_ – (Ct_target_ – Ct_control_)_CH_ breed. Generalized linear model was used to test the comparisons among breed genotypes adjusted with gender effect.

## RESULTS

### Abdominal, subcutaneous, and intramuscular fat deposition

The growth performance of crossbreed chickens was increased by the commercial breed fraction and growth performance including body weight, feed intake and feed conversion ratio of the various breed chicken in this study are displayed in Table 2. Fat deposition in both abdominal fat and skin percentage of various breed fraction chickens at 6 to 12 weeks of age is shown in Table 3. At 6 weeks of age, the abdominal fat and skin percentage of commercial broiler chickens were higher than the CH, KMI, and KM2 breeds (p<0.05). From 8 to 12 weeks of age the KM2 breed had a higher fat deposition in abdominal fat than KM1 and CH breeds (p< 0.05). The total skin percentage was higher in the BR breed compared to all the other breeds at 6 weeks of age, however, there was no difference between the crossbreeds, but both crossbreeds had higher percentage total skin percentage (p<0.05) than the CH breed. From week 8 to 12, there were no differences between the crossbreeds with respect to total skin percentage, however, both crossbreeds had higher percentage total skin compared to the CH breed. At 6 weeks of age, there as a significant difference (p<0.05) in *P. major* intramuscular fat between the CH and BR breeds, but no difference between the two crossbreeds. Between 8 to 12 weeks of age, the percentage intramuscular fat was significantly lower (p<0.05) in the CH compared to the crossbreeds. There was no difference in intramuscular fat between the crossbreeds.

### Transcriptional level of fatty acid binding proteins in abdominal fat

**Figure 1:**
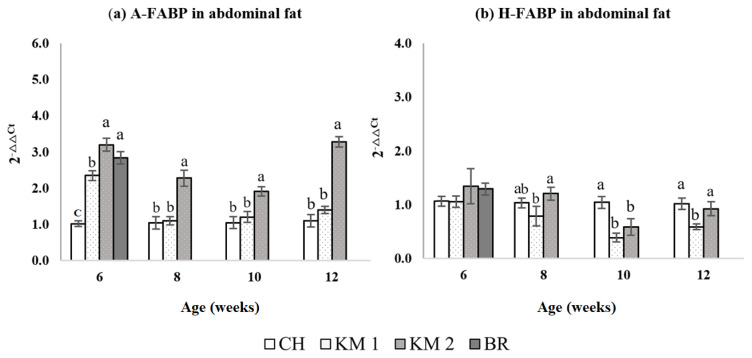
Comparison of adipocyte type fatty acid binding protein (A-FABP) (a) and heart type FABP (H-FABP) (b) relative transcriptional expression level in abdominal fat tissue. CH, Chee breed (100% Thai native chicken background: 0% broiler background); KM1, Kaimook e-san1 (50% Thai native chicken background: 50% broiler background); KM2, Kaimook e-san2 (25% Thai native chicken background: 75% broiler background); BR, broiler chicken (0% Thai native chicken background: 100% broiler background). ^a–c^ Mean values within a column with no common superscript differ significantly (p<0.05).

The relative mRNA expressions of A-FABP and H-FABP for abdominal fat percentage at the different ages of slaughter are presented in Figure 1. The result of A-FABP fold change (Figure 1a) demonstrated that at 6 weeks, CH had a significantly lower (p<0.05) expression of A-FABP compared to the other breeds. There was no difference in mRNA expression of A-FABP between KM2 and BR (p>0.05), however, the expression of KM1 was higher than CH, but lower than both KM2 and BR. From 8 to 12 weeks, the KM2 breed had a significantly high expression (p<0.05) compared to the KM1 and CH breeds. The mRNA expression of H-FABP was not different between breeds at 6 weeks (Figure 1b). At 8 weeks, the expression in KM2 was higher than KM1, but not different from CH. At week 10, the highest expression of H-FABP was observed in CH compared to KM1 and KM2, and there was no difference (p>0.05) in expression between KM1 and KM2. At 12 weeks of age, the H-FABP expression of KM1 was significantly lower (p<0.05) than that of CH and KM2, and that of CH and KM2 was not different (p>0.05) (Figure 1b).

### Transcriptional level of fatty acid binding proteins in breast muscle (intramuscular fat)

**Figure 2:**
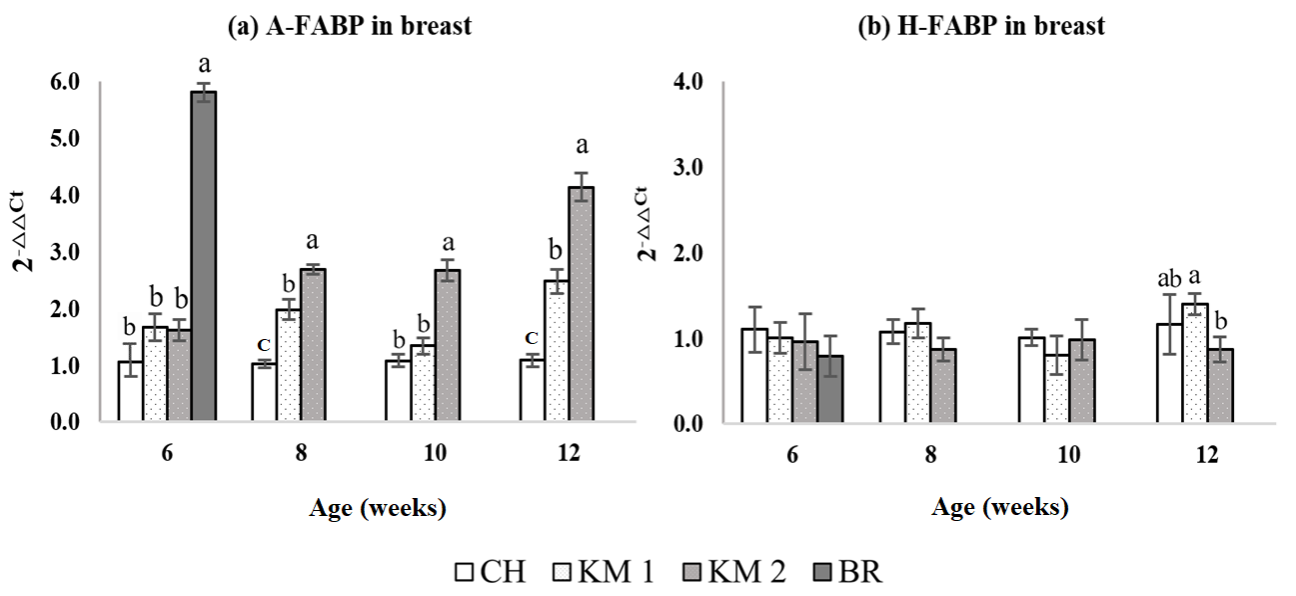
Comparison of adipocyte type fatty acid binding protein (A-FABP) (a) and heart type FABP (H-FABP) (b) relative transcriptional expression level in breast muscle tissue. CH, Chee breed (100% Thai native chicken background: 0% broiler background); KM1, Kaimook e-san1 (50% Thai native chicken background: 50% broiler background); KM2, Kaimook e-san2 (25% Thai native chicken background: 75% broiler background); BR, broiler chicken (0% Thai native chicken background: 100% broiler background). ^a–c^ Mean values within a column with no common superscript differ significantly (p<0.05).

The mRNA expressions of A-FABP and H-FABP in *P. major* intramuscular fat are shown in Figure 2. At 6 weeks, the mRNA expression of A-FABP in intramuscular fat was significantly higher (p<0.05) in the BR chickens compared with the CH, KM1, and KM2 chickens, however, there were no differences among the CH, KM1, and KM2 chickens. The expression of A-FABP in the intramuscular fat for KM2 was consistently higher than CH and KM1 from 8 to 12 weeks. The A-FABP expression in the KM1 chickens appeared to be intermediate between the CH and KM2 groups from 8 to 12 weeks. There were no differences in the expression of H-FABP among the breeds from 6 to 10 weeks. At 12 weeks, H-FABP expression was significantly reduced (p<0.05) in the KM2 birds compared with the KM1 birds, however, there was no difference in expression between the KM1 and CH birds.

## DISCUSSION

### Effect of Thai native genetic background on carcass fat deposition

Abdominal fat is an excess fat considered as a waste in the slaughtering process, a major part of lipid accumulation in chickens [5], and represents about 10% to 15% of carcass weight [16]. Abdominal fat has been shown to have a positive genetic correlation with total carcass lipid [17]. In the current study, we show that the CH breed had the lowest abdominal fat and abdominal fat percentage which increased with increased broiler background in the genotype. This clearly shows a positive relationship between growth rate and abdominal fat percentage. Thus, crossbreeding of the CH breed with exotic breeds like the broiler can increase productivity by improving growth rate, but can concomitantly lead to increased abdominal fat. This is in concordance with the study of Yin et al [18]. However, several layer crossbred sires comprising of layer×broilers, layer×Shanghai, and layer×Shanghai Road Bar when crossed with the Chee dam produced progeny with similar abdominal fat percentage [19]. Crossing Indonesian native chickens did not change the abdominal fat percentage among the crossbreed progenies [20]. In the current study, both KM1 and KM2 had high abdominal fat percentages compared to CH, however, KM2 accumulated abdominal fat at a higher rate than KM1 over the course of the study. The slaughtering age of broiler×CH crossbreeds can be manipulated to strike the right balance between growth rate and accumulation of abdominal fat.

It has been reported that abdominal fat is positively correlated with skin fat percentage [5]. In the current study, the CH breed had the lowest skin percentage, but despite the high abdominal fat differences between KM1 and KM2, there were no differences in skin percentage. The lack of difference in skin percentage between KM1 and KM2 may be due to the influence of the CH breed.

### Regulation of fatty acid binding proteins gene on fat deposition in adipose tissue and intramuscular fat

In chickens, a large proportion of the adipose tissue is stored in the abdomen as abdominal fat. In the current study A-FABP expression level at 6 weeks of age correlated with the abdominal fat levels. The mRNA expression of A-FABP was highest in BR and KM2 breeds and lowest in CH. The abdominal fat percent of the KM1 breed was intermediate between the CH and, KM2 breed. The *A-FABP* gene is expressed abundantly in mature adipocytes of chickens [6,10]. Thus, the mRNA expression level is related to the amount of abdominal fat present. The expression of A-FABP may also be affected by the genotype and age. From 8 to 12 weeks, there was an increased expression of A-FABP in KM2 compared to KM1 and CH. Even though KM1 had 50% broiler background with a higher percentage of abdominal fat compared with CH, the expression levels of A-FABP were similar.

The role of A-FABP cellular fat deposition mechanism in chickens may be similar to that in mammalian species, such as mice [7], cattle [21], and swine [22]. A-FABP plays a key role in cellular transport of fatty acid and accumulation of triglycerides [10], especially with the high affinity for long chain fatty acid [22]. In cattle, Michal et al [21] reported that expression of A-FABP is highly related to subcutaneous fat deposition. Likewise, our study found that subcutaneous fat is associated with quantity of A-FABP.

Intramuscular fat is one of the most important meat quality traits [21,22]. The level of intramuscular fat in indigenous chicken meat makes them desirable over the exotic broiler breeds [23,24]. The A-FABP gene is widely expressed in in skeletal muscle, stomach and lung tissues [24]. In the current study, A-FABP expression level in the *P. major* was significantly higher in BR compared with all the other breeds at 6 weeks. Even though there was a phenotypic difference between KM2 and CH regarding intramuscular fat, the expression of A-FABP was the same. From 8 to 12 weeks, there appears to be a relationship between intramuscular A-FABP expression and genetic background. Intramuscular fat is more important with respect to meat quality and value in beef cattle and swine than chickens. In pigs, Chen et al [25] reported that expression of A-FABP in intramuscular fat increased with age. Genetic markers in A-FABP have been found to associate with intramuscular fat in Chinese indigenous chicken [26]. If such genetic markers exist in the CH breeds and CH×exotic crossbreeds, it could be incorporated into a breeding program to modulate the accumulation of both visceral and intramuscular fat in these breeds.

H-FABP is a protein related to intracellular transportation of fatty acid from the plasmalemma to the site of β-oxidation in mitochondria [8,9]. H-FABP is also related to fatty acid intake and adipogenic differentiation and utilization of fat [8,27]. H-FABP is distributed in many tissues that have a high energy consumption by the activity of fatty acid oxidation., such as skeletal muscle and cardiac muscle because it participates in transporting of fatty acid [28]. In the current study, there was no consistent pattern of H-FABP expression in both abdominal fat and intramuscular fat. H-FABP expression has been suggested to adjust fatty acid activity and produce energy from fat sources to meet the requirement of animals [24]. Shi et al [12] suggested that the differentiation and development of adipocyte cells in fatty tissue involves several pathways. Therefore, the regulation of adipocyte growth may be dependent on a cascade of genes and several FABP including A-FABP and H-FABP. Wang et al [24] reported the negative correlation between H-FABP mRNA and intramuscular fat in an indigenous chicken breed. However, we did not observe such correlation in the current study.

## CONCLUSION

We studied the abdominal fat percentage and skin percentage of indigenous Thai chickens and their crossbreeds with different degrees of broiler chicken background. The amount of fat deposition directly correlated with the genetic background. The native CH accumulated the least amount of fat compared with KM1 and KM2 which are crossbreeds of the CH with 50% and 75% broiler background, respectively. Accordingly, the mRNA expressions of A-FABP in the abdominal fat and intramuscular fat of the *P. major* were highest in the broiler and lowest in the CH at 6 weeks of age. From 8 to 12 weeks of age, mRNA expression levels of A-FABP in both tissues was dictated by the genetic background, where the CH was lowest, followed by KM1, then KM2. Thus, the expression level of A-FABP may correlate well with accumulation of visceral, subcutaneous fat and intramuscular fat. There was no consistent pattern of expression of H-FABP across age and breed. It is thought that since FABPs are involved in fat deposition, genetic markers in these genes could be used in marker assisted studies to select against excessive fat accumulation.

## Figures and Tables

**Table 1:** Primer sequence, polymerase chain reaction product size and annealing temperature

Genes	Sequences	Product (bp)	TM	Source
*H-FABP*	F: 5-CAGAAGTGGGATGGGAAGGAGA-3	104	60	[24]
	R: 5-TCATAGGTGCGGGTGGAGAC-3			
*A-FABP*	F: 5-ATGTGCGACCAGTTTGT-3	143	54	[29]
	R: 5-TCACCATTGATGCTGATAG-3			
*18S rRNA*	F: 5-CGGCGACGACCCATTCGAAC-3	99	62	[30]
	R: 5-GAATCGAACCCTGATTCCCCGTC-3			

TM, primer melting temperature; *H-FABP*, heart type fatty acid binding protein; *A-FABP*, adipocyte type fatty acid binding protein.

**Table 2:** Body weight, feed intake and feed conversion ratio (from day old chick to 6th, 8th, 10th, and 12th respectively) in different Thai native chickens, commercial broilers and their crossbreed genotypes1) at different ages of chickens

Traits	Breeds	Age of slaughter (wk)			

		6	8	10	12
Body weight (g)	CH	447.1^d^	665.2^c^	906.6^c^	1,160.1^c^
	KM1	621.0^c^	895.7^b^	1,231.2^b^	1,500.5^b^
	KM2	1,101.9^b^	1,629.4^a^	2,145.4^a^	2,680.9^a^
	BR	2,263.9^a^	-	-	-
Feed intake (g)	CH	1,379.3^c^	1,981.8^c^	2,698.4^c^	3,594.7^c^
	KM1	1,404.4^c^	2,254.5^b^	3,279.1^b^	4,257.6^b^
	KM2	2,247.8^b^	3,653.5^a^	5,281.8^a^	7,070.6^a^
	BR	4,221.2^a^	-	-	-
Feed conversion ratio	CH	3.08^a^	3.12^a^	3.08^a^	3.18^a^
	KM1	2.41^b^	2.63^b^	2.75^b^	2.91^b^
	KM2	2.10^c^	2.29^c^	2.50^c^	2.68^c^
	BR	1.90^d^	-	-	-

1) CH, Chee breed (100% Thai native chicken background: 0% broiler background); KM1, Kaimook e-san1 (50% Thai native chicken background: 50% broiler background); KM2, Kaimook e-san2 (25% Thai native chicken background: 75% broiler background); BR, broiler chicken (0% Thai native chicken background: 100% broiler background).

a–d Mean values within a column with no common superscript differ significantly (p<0.05).

**Table 3:** Percentage fat deposition in different Thai native chickens, commercial broiler and their crossbreed genotypes1) at different ages of slaughter

Traits	Breeds1)	Age of slaughter (wk)			

		6	8	10	12
Abdominal fat (%)	CH	0.35^d^	0.28^c^	0.25^c^	0.24^c^
	KM1	1.20^c^	1.22^b^	1.47^b^	1.87^b^
	KM2	2.23^b^	2.50^a^	3.18^a^	3.93^a^
	BR	2.53^a^	-	-	-
	SEM	0.127	0.159	0.197	0.170
	p-value	0.001	0.001	0.001	0.001
Total skin (%)	CH	6.35^c^	6.53^b^	6.56^b^	6.55^b^
	KM1	7.73^b^	7.59^a^	7.87^a^	8.63^a^
	KM2	7.82^b^	7.86^a^	8.25^a^	8.56^a^
	BR	8.40^a^	-	-	-
	SEM	0.238	0.312	0.254	0.261
	p-value	0.001	0.009	0.001	0.001
Intramuscular fat (%)(Breast muscle)	CH	1.04^c^	1.11^b^	1.18^b^	1.17^b^
	KM1	1.24^bc^	1.26^ab^	1.26^b^	1.47^ab^
	KM2	1.52^ab^	1.53^a^	1.65^a^	1.93^a^
	BR	1.76^a^	-	-	-
	SEM	0.151	0.128	0.121	0.162
	p-value	0.032	0.038	0.019	0.015

SEM, standard error of the mean.

1) CH, Chee breed (100% Thai native chicken background: 0% broiler background); KM1, Kaimook e-san1 (50% Thai native chicken background: 50% broiler background); KM2, Kaimook e-san2 (25% Thai native chicken background: 75% broiler background); BR, broiler chicken (0% Thai native chicken background: 100% broiler background).

a–d Mean values within a column with no common superscript differ significantly (p<0.05).
